# Multienzyme Platform for the Synthesis of UDP Sugars and Human Milk Oligosaccharides

**DOI:** 10.1002/cbic.202500716

**Published:** 2026-04-22

**Authors:** Tuan Son Hoang, Fabian Lange, Sebastian Bruno Kleeberg, Lea Thomas, Nam-Hai Hoang, Udo Reichl, Thomas F. T. Rexer

**Affiliations:** ^1^ Department of Bioprocess Engineering Max Planck Institute for Dynamics of Complex Technical Systems Magdeburg Germany; ^2^ Chair of Bioprocess Engineering Otto‐von‐Guericke University Magdeburg Magdeburg Germany; ^3^ eversyn GmbH Magdeburg Germany

**Keywords:** cell‐free biocatalysis, human milk oligosaccharides, multienzyme cascade, nucleotide sugar regeneration, UDP‐GlcNAc and UDP‐Gal

## Abstract

Naturally occurring in breast milk, human milk oligosaccharides (HMOs) are of great interest as an ingredient for infant nutrition due to numerous associated health benefits. Current commercial production relies mainly on microbial fermentation, while enzymatic synthesis is used to produce milligram scales for scientific studies. Enzymatic synthesis using glycosyltransferases and nucleotide sugars is especially promising due to high reaction yields, but is limited by low activity of glycosyltransferases and the high cost of nucleotide sugars. This study presents a novel approach that uses a single engineered *E. coli* BL21(DE3) strain to simultaneously express six recombinant enzymes (UMPK, PPK3, GALK, NAHK, GALU, and PPA). This enables dual‐nucleotide sugar synthesis through two integrated multienzyme cascades. The system uses cost‐effective substrates, including uridine 5′‐monophosphate (UMP), *N*‐acetylglucosamine (GlcNAc), galactose (Gal), and ATP. In situ ATP regeneration is achieved through polyphosphate (PolyP_*n*_) breakdown. Comparative studies of three different expression strain configurations demonstrated that crude cell lysate could serve as an effective biocatalyst. This eliminates the need for expensive enzyme purification while maintaining high catalytic activity. Using crude cell lysate, conversion yields approaching 100% were obtained. Both UDP‐GlcNAc and UDP‐Gal were successfully purified using anion‐exchange chromatography. Based on the UV spectrum, purities of 85–99% and recovery yields exceeding 90%, respectively, were achieved. The practical application of this system was demonstrated by the successful synthesis of two HMOs: Lacto‐*N*‐triose II (LNTII) and lacto‐*N*‐neotetraose (LNnT), which demonstrates an effective nucleotide sugar recycling in coupled enzymatic reactions and paves the way toward larger scale production.

## Graphical abstract

Engineered *Escherichia coli* produces six enzymes (UMPK, PPK3, GALK, NAHK, GALU, PPA) that are purified and used in a cell‐free cascade to synthesize UDP‐*N*‐acetylglucosamine (UDP‐GlcNAc) and UDP‐galactose (UDP‐Gal) from inexpensive substrates. The cascades can be coupled with glycosyltransferases for in situ nucleotide sugar regeneration, enabling oligosaccharide synthesis, including human milk oligosaccharides.

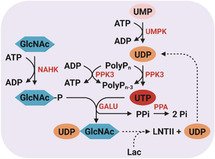

## 1 Introduction

Human milk oligosaccharides (HMOs) are of great interest in infant formula production due to the numerous associated health benefits, including support for gut microbiome balance, intestinal barrier function, immune, and brain development [1, 2, 3]. Clinical studies on the supplementation of biotechnologically manufactured HMOs found that they are safe and well‐tolerated by infants [4]. Driven by significant academic interest in structural and functional elucidation, around 200 distinct HMOs structures have been identified in human milk to date [5, 6, 7]. The most common HMOs in infant formula currently include lacto‐*N*‐neotetraose (LNnT), 3′‐sialyllactose (3′‐SL), 6′‐sialyllactose (6′‐SL), and 2′‐fucosyllactose (2′‐FL) [8, 9]. In general, HMOs can be produced by chemical, cell‐free enzymatic, or whole‐cell approaches [10]. The latter is the most widely used method for the commercial production of HMOs. However, the efficient synthesis of complex HMOs of more than four monosaccharide units is difficult due to side reactions within the cells and transport limitation across cell membranes [11]. Chemical synthesis faces numerous drawbacks that hinder its use in commercial production. These include high production costs, low yields, and the possibility of side reactions. Moreover, the multistep processes are environmentally harmful, with many reactants even being prohibited in the food industry [12]. For this reason enzymatic synthesis is becoming increasingly important, as it offers high stereoselectivity, regioselectivity, yield, and purity [8, 13]. In general, two enzymatic approaches have been used to synthesize HMOs: the transglycosylation approach where monosaccharides are transferred from one glycan to another and synthesis using glycosyltransferases where nucleotide sugars serve as glycan building blocks [2, 14]. In comparison to the former, glycosyltransferase‐catalyzed reactions typically show high conversion yields and eliminate the need to separate HMO mixtures in downstream processing operations. The lactose core structure of HMOs can typically be extended with activated sugars UDP‐Gal, UDP‐GlcNAc, GDP‐fucose, and CMP‐Neu5Ac through various Leloir glycosyltransferases [13]. Due to the high cost and low availability of the nucleotide sugars, enzymatic synthesis of HMOs using glycosyltransferases is not yet economically viable [15].

Although there are already many enzymatic approaches for the synthesis of nucleotide sugars, chemical synthesis is still widely used [16, 17]. However, this approach typically involves multiple steps, low yields, low stereoselectivity, and requires protecting groups, all of which contribute to high production costs [18, 19]. To counteract these disadvantages, enzymatic synthesis offers a good alternative, which typically involves one‐pot reactions through multienzyme cascades, utilizing affordable and readily available precursors [20, 21]. These approaches enable simplified processes, reduce costs, and increase efficiency while maintaining ecofriendly conditions [17, 22]. So far, UDP‐GlcNAc and UDP‐Gal have been enzymatically synthesized with percentage yields approaching 100% [23, 24]. Yet, many current synthesis routes depend on expensive substrates, such as UTP and ATP, and require purified enzymes as biocatalysts, limiting their scalability to the liter scale [25, 26].

In this study, an *E. coli* strain was used to simultaneously express six recombinant enzymes for the synthesis of UDP‐GlcNAc and UDP‐Gal based on the well‐established cascades of Mahour et al. [27, 28]. Using one single strain, the biocatalyst required for both reactions can be produced, reducing the need for numerous enzyme expressions and purifications. The two multienzyme cascades enable the nucleotide sugar synthesis from the inexpensive precursors UMP, GlcNAc, or Gal and ATP. The latter is regenerated using inexpensive PolyP_*n*_. To demonstrate how the synthesis of nucleotide sugars can be integrated into HMO synthesis, two concepts are demonstrated, the cascade in situ coupled with a glycosyltransferase and utilizing semipurified UDP‐Gal.

## 2 Results and Discussion

### 2.1 Multienzyme Cascade Design

**FIGURE 1:**
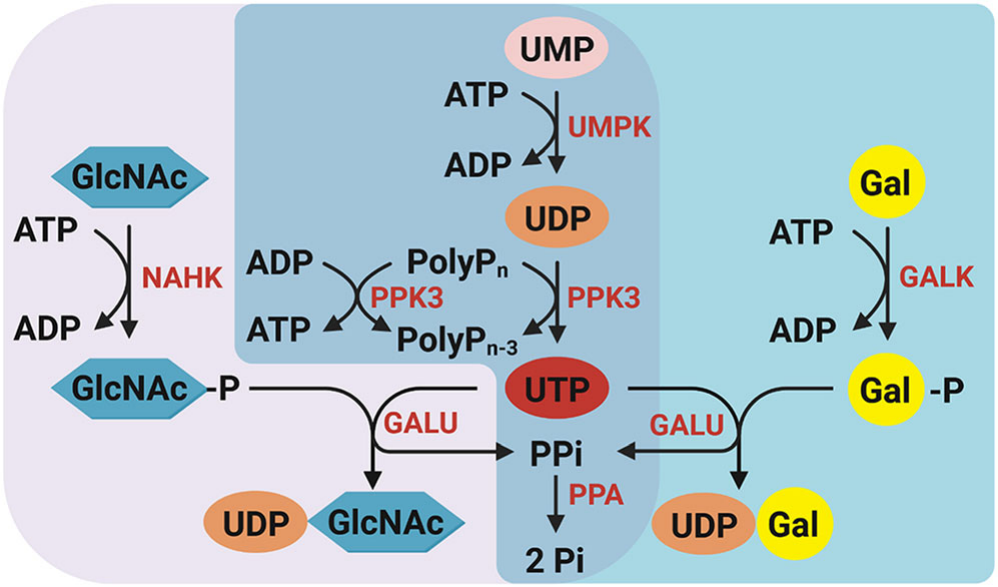
The illustration of two multienzyme cascades expressed in one *E. coli* strain for the synthesis of the nucleotide sugars UDP‐GlcNAc and UDP‐Gal. The UDP‐GlcNAc synthesis pathway is highlighted in violet and the UDP‐Gal pathway in blue.

For the synthesis of UDP‐GlcNAc and UDP‐Gal, two multienzyme cascades based on the study by Mahour et al. [27, 28] were adapted (Figure 1 & Table 1). The enzyme sequences were taken from UniProt database. Starting from UMP as substrate, the enzyme UMP‐CMP kinase (UMPK, O04905) catalyzes the phosphorylation of UMP to UDP at the expense of ATP. In the next step the enzyme polyphosphate kinase (PPK3, Q5LSN8) converts UDP to UTP by hydrolysis of PolyP_*n*_. Using GlcNAc as substrate, the enzyme *N*‐acetylhexosamine 1‐kinase (NAHK, E8MF12) phosphorylates GlcNAc to GlcNAc‐1P by ATP consumption. In contrast, the enzyme galactokinase (GALK, B3DTF0) is needed when Gal is applied as a substrate. Gal is converted to Gal‐1P with the consumption of ATP. Depending on the sugar substrate, the enzyme UTP‐glucose‐1‐phosphate uridylyltransferase (GALU, P0AEP3) forms UDP‐GlcNAc or UDP‐Gal by combining UTP with GlcNAc‐1P or Gal‐1P, respectively. During this reaction, the inhibitory diphosphate is formed, which is then cleaved by the enzyme inorganic pyrophosphatase (PPA, P57918) into monophosphates. This process also shifts the equilibrium toward nucleotide sugar synthesis.

**TABLE 1:** List of enzymes used in this study for the synthesis of UDP‐GlcNAc and UDP‐Gal in the multienzyme cascades.

Enzyme	Name	Uniprot‐ID	EC No.	Catalyzed reaction	Molecular weight, kDa	Origin
**GALK**	Galactokinase	B3DTF0	2.7.1.6	Gal + ATP → Gal‐1P + ADP	44.3	*Bifidobacterium longum*
**GALU**	UTP‐glucose‐1‐phosphate uridylyltransferase	P0AEP3	2.7.7.9	UTP + GlcNAc/Gal‐1P → UDP‐GlcNAc/Gal + PPi	32.9	*Escherichia coli* K12
**NAHK**	*N*‐acetylhexosamine 1‐kinase	E8MF12	2.7.1.162	GlcNAc + ATP → GlcNAc‐1P + ADP	39.9	*Bifidobacterium longum*
**PPA**	Inorganic pyrophosphatase	P57918	3.6.1.1	PPi → 2 Pi	19.3	*Pasteurella multocida*
**PPK3**	Polyphosphate kinase	Q5LSN8	2.7.4.1	PolyP_*n*_ + NDP → PolyP_n−1_ + NTP	34.7	*Ruegeria pomeroyi*
**UMPK**	UMP‐CMP kinase	O04905	2.7.4.14	UMP + ATP → UDP + ADP	22.5	*Arabidopsis thaliana*

To enable the simultaneous expression of all six enzymes in *E. coli* BL21(DE3) for the multienzyme cascades, various combined duet expression vectors (Sigma–Aldrich, USA) were used (Table 2).

**TABLE 2:** Plasmid and enzyme combinations of different expression strains.

Strain A	Strain B	Strain C
pACYCDuet UMPK/PPK3	pRSFDuet PPK3/GALU	pACYCDuet UMPK/PPK3
pCDFDuet GALU/PPA	pCDFDuet UMPK/PPA	pCDFDuet GALU/PPA
pRSFDuet GALK/NAHK	pETDuet GALK/NAHK	pETDuet GALK/NAHK

The purified enzyme mixtures from all three expression strains exhibit relatively high purity > 70% (Figure SI 9). However, the enzyme mixture of strain C shows additional protein bands below 25 kDa and above 50 kDa, which do not match the expected sizes of the target proteins, indicating impurities. In general, the target proteins of both cascades (Figure 1) are present in all enzyme mixtures (Figure SI 9). However, due to the overlapping molecular weights of NAHK, GALU, and PPK3 (35–40 kDa), these enzymes are difficult to distinguish within the mixtures.

All strains exhibit comparable expression levels of the recombinant proteins (Table 3). Strain B shows the highest expression with 0.14 g/L, followed by strains C and A with 0.11 g/L and 0.09 g/L, respectively. The expression levels observed in this study are comparable with values reported in the literature, where recombinant protein yields in the milligram range per liter of culture are common [29, 30]. However, some studies have also reported yields in the gram‐per‐liter range [31].

**TABLE 3:** Overview of the expression and purification of enzyme mixtures and crude cell lysates. *V* is the total volume of the biocatalyst stock solution. Conc. is the total protein concentration of the biocatalyst stock solution. The mass refers to the total protein mass of each biocatalyst stock solution.

Expression strain	Enzyme formulation	***V*, mL**	Conc., g/L	Mass, mg	Expression volume, mL	**Expression** level, g/L
Strain A	purified enzyme mixture	6	3.10	18.58	200	0.09
	crude cell lysate	45	4.37	196.63	200	
Strain B	purified enzyme mixture	4.5	6.11	27.49	200	0.14
	crude cell lysate	30	6.92	207.65	200	
Strain C	purified enzyme mixture	4.1	5.25	21.53	200	0.11
	crude cell lysate	45	6.74	303.21	200	

### 2.2 Proof of Concept

In the following experiments, the purified enzyme compositions from the three generated strains are referred to as (purified) enzyme composition A, B, and C. The conversion/percentage yield (%) is defined as the ratio of the UDP‐GlcNAc/Gal molarity to the initial UMP molarity. In an initial set of reactions, the enzyme compositions A, B, and C were tested for the synthesis of UDP‐GlcNAc and UDP‐Gal. Synthesis reactions were performed in batch mode at 37°C for 24 h with 1 mM UMP, 1 mM GlcNAc/Gal in aqueous buffered solution with 150 mM Tris‐HCl (pH 7.5).

**FIGURE 2:**
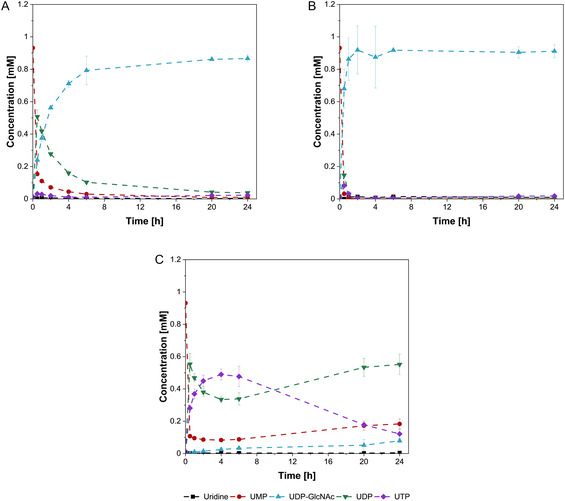
Time courses of uridine‐based intermediates in the UDP‐GlcNAc synthesis. Trial with purified enzyme mixtures of the different expression strains. (A) Enzyme mixture of strain A. (B) Enzyme mixture of strain B. (C) Enzyme mixture of strain C. The experimental conditions: 150 mM Tris‐HCl (pH 7.5), 75 mM MgCl_2_, 1 mM UMP, 1 mM GlcNAc, 2.5 mM ATP, 2 mM PolyP_*n*_, 1 g/L total protein concentration in a total volume of 500 μL. Incubation for 24 h at 37°C and 500 rpm. Enzymatic reactions were performed in biological triplicate, mean and standard deviation.

All three compositions were capable of synthesizing UDP‐GlcNAc (Figure 2). Enzyme composition C obtained the lowest final titer of approximately 0.08 mM UDP‐GlcNAc. In contrast, compositions A and B achieved significantly higher final titers of 0.87 and 0.91 mM, respectively. Considering the error of the time course, only composition B achieved almost complete conversion within 2 h, indicating higher productivity compared to composition A and C.

**FIGURE 3:**
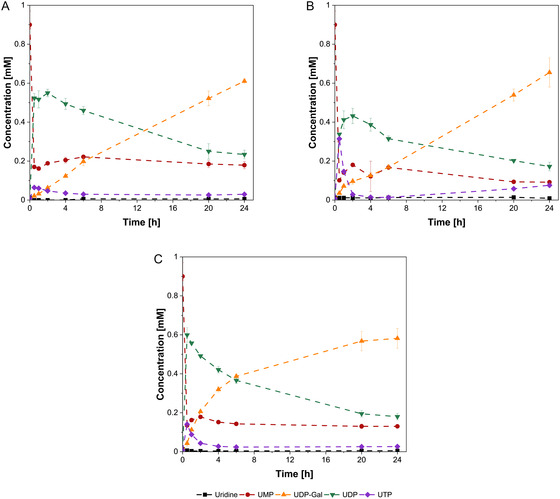
Time courses of uridine‐based intermediates in the UDP‐Gal synthesis. Trial with purified enzyme mixtures of the different expression strains. (A) Enzyme mixture of strain A. (B) Enzyme mixture of strain B. (C) Enzyme mixture of strain C. The experimental conditions: 150 mM Tris‐HCl (pH 7.5), 75 mM MgCl_2_, 1 mM UMP, 1 mM Gal, 2.5 mM ATP, 2 mM PolyP_*n*_, 1 g/L total protein concentration in a total volume of 500 μL. Incubation for 24 h at 37°C and 500 rpm. Enzymatic reactions were performed in biological triplicate, mean and standard deviation.

UDP‐Gal synthesis reactions with the different enzyme compositions yielded similar final titers after 24 h, ranging from 0.58 to 0.66 mM UDP‐Gal (Figure 3). Enzyme composition B achieved the highest final titer at 0.66 mM, followed by composition A at 0.61 mM, while both exhibit a similar UDP‐Gal progression throughout the reaction. The reaction of enzyme composition C exhibited the highest productivity during the first 6 h of reaction time, after which the reaction reached equilibrium, yielding a final titer of 0.58 mM.

Overall, activity tests for UDP‐GlcNAc and UDP‐Gal synthesis (Figures 2 and 3), combined with SDS‐PAGE analysis, confirmed that all six enzymes involved in both cascades were successfully coexpressed and functionally active. According to the results, the choice of plasmid and varying combinations resulted in different compositions of the enzyme mixtures, leading to different performance in the synthesis of nucleotide sugars (Figure 2 and 3). For the synthesis of the nucleotide sugars UDP‐GlcNAc and UDP‐Gal, only a small difference in titer and productivity was observed between enzyme compositions A and B. Enzyme composition C exhibited substantially lower UDP‐GlcNAc synthesis rates and was consequently excluded from further analysis. The underlying cause remains unclear from the present data and a more detailed analysis including for example proteomic quantification of enzyme concentrations is necessary to elucidate the underlying mechanism.

Moreover, the synthesis rate of UDP‐Gal (strain A‐C) is significantly slower than that of UDP‐GlcNAc (strain A and B). This is due to the different pH optima of the enzymes GALK and NAHK. The pH optimum of the latter, at pH 8.5 [32], is closer to the initial reaction pH of 9 while GALK assumed to prefer lower pHs at 6–7.5 [33, 34]. Consequently, GALK is the rate limiting enzyme as evident from the accumulation of UMP/UDP/UTP (Figure 3).

### 2.3 Synthesis at Increased Substrate Concentrations

Crude cell lysates of strains A and B, along with the corresponding purified enzyme compositions, were evaluated for UDP‐GlcNAc and UDP‐Gal synthesis at varying substrate molarities. Synthesis reactions were performed at 50/70/100 mM UMP and 50/70/100 mM GlcNAc/Gal in 150 mM Tris‐HCl (pH 9).

**FIGURE 4:**
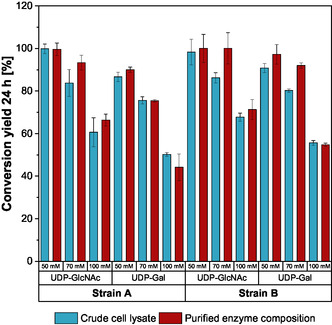
Comparison of the conversion yields of different expression strains for the UDP‐GlcNAc and UDP‐Gal synthesis at various substrate levels at 50, 70, and 100 mM UMP and GlcNAc/Gal, respectively. Enzymatic reactions were performed in biological triplicate, mean and standard deviation.

A comparison of expression strains A and B showed that both biocatalyst formulations of strain B consistently outperformed those of strain A, resulting in higher conversion yields after 24 h reaction time (Figure 4). Moreover, purified enzyme composition as biocatalyst generally achieved higher conversion yields than the corresponding crude cell lysate in most reactions.

The synthesis of UDP‐GlcNAc resulted overall in higher conversion yields compared to UDP‐Gal synthesis. However, at substrate molarities of 50 mM and 70 mM, complete conversion was achieved in several reactions for both UDP‐GlcNAc and UDP‐Gal synthesis (Figure 4). In general, increasing substrate molarities resulted in lower percentage yields. In contrast to synthesis reactions at 50 mM substrate concentrations, reactions at 70 mM and 100 mM rarely achieved complete conversion, with most approaching equilibrium within 7–20 h (Figures S1 and S2). As demonstrated in previous work, this is likely due to pH drop caused by polyphosphate conversion and, thus, loss of enzyme activity [20, 21, 28]. However, maximum final titers of up to 71 mM UDP‐GlcNAc (43 g/L) and 62 mM UDP‐Gal (35 g/L) could be achieved using purified enzyme compositions. In contrast, final titers of up to 66 mM UDP‐GlcNAc (37 g/L) and 52 mM UDP‐Gal (30 g/L) could be achieved using crude cell lysate.

Further, screening at higher substrate concentrations revealed that in general, biocatalyst formulations of strain B achieved significantly higher conversion and productivities compared to strain A (Figure 4). This highlights the significant impact of vector selection and enzyme distribution across multiple cloning sites on the activity of individual enzymes and consequently, on the performance of the entire enzyme cascade [35, 36, 37]. When comparing crude cell lysate and purified enzyme composition, the differences in conversion yield and the overall performance were relatively small, although the ratio of recombinant proteins in the lysates is significantly lower than in the purified enzyme compositions due to the presence of host cell proteins (Figure SI 9). These results show that the use of crude cell lysates instead of purified enzyme composition as biocatalyst proved to be a feasible alternative (Figure 4), with the crude cell lysate of strain B showing the greatest potential for achieving higher titers and conversion yields. Consequently, enzyme purification is not required, leading to an up to fivefold reduction in supply costs of the biocatalyst [38].

### 2.4 Chromatographic Purification of UDP‐GlcNAc & UDP‐Gal

**FIGURE 5:**
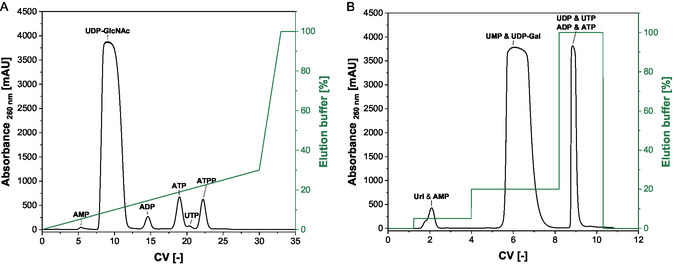
UV spectra of the reaction samples after purification by anion‐exchange chromatography. (A) UDP‐GlcNAc was fractionated from 7.5 CV to 12.5 CV. (B) UDP‐Gal was fractionated from 5.5 CV to 8 CV. Equilibration buffer: 1 mM NaOH, elution buffer: 1 mM NaOH with 1 M NaCl, column volume 5 mL, flowrate 5 mL/min.

For the purification of UDP‐GlcNAc and UDP‐Gal, anion‐exchange chromatography was performed using the gradients shown in Figure 5.

Typically, a total of 28.2 mg UDP‐GlcNAc with a purity of 95% in the UV spectrum was loaded onto the anion‐exchange column. Fractions with a total amount of 26.1 mg UDP‐GlcNAc could be purified at a purity of >99% with regard to the UV‐active components (Figure S3), resulting in a recovery yield of 93%. In some synthesis reactions resulting in a complete conversion, a modified form of the cosubstrate ATP ATPP was found, as described by Mordhorst, et al. [39]. This intermediate could be discovered and separated using the purification method. However, UDP‐Gal was purified in comparable quantities with a purity of > 85% with respect to the UV‐active components (Figure SI 4) and a recovery rate exceeding 90%. The ^1^H and ^13^C‐NMR spectra further confirmed the structure and purity of the produced compounds (Figure SI 5–8).

### 2.5 Synthesis of HMOs – Cascade Coupling and Semipurification

This section demonstrates that the cascade enables nucleotide sugar recycling for HMO synthesis and that, even from using crude cell lysate, simple ultrafiltration generates UDP‐Gal pure enough for HMO synthesis.

#### 2.5.1 LNTII

**FIGURE 6:**
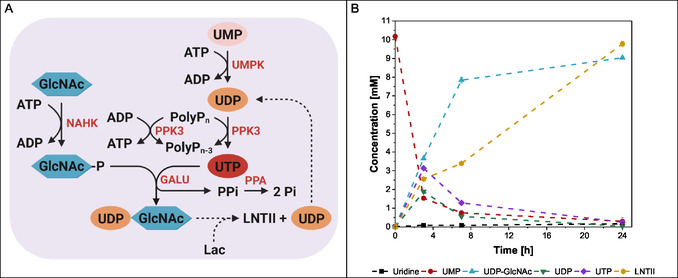
(A) Illustration of the UDP‐GlcNAc regeneration system for LNTII synthesis. Dashed arrows indicate the pathway added to integrate the LgtA enzyme into the UDP‐GlcNAc synthesis cascade. (B) Experimental conditions: 150 mM Tris‐HCl (pH 9), 150 mM MgCl_2_, 10 mM UMP, 50 mM GlcNAc, 100 mM lactose, 2.5 mM ATP, 45 mM PolyP_*n*_, 1 g/L crude cell lysate, and 0.2 g/L LgtA. Incubation for 24 h at 37°C and 300 rpm. Enzymatic reactions were performed in biological triplicate, mean and standard deviation.

The one‐pot synthesis of LNTII was performed by coupling an enzymatic cascade for UDP‐GlcNAc generation with the glycosyltransferase LgtA‐catalyzed transfer of GlcNAc to lactose to demonstrate the cascade can efficiently recycle nucleotide sugars for HMO synthesis (Figure 6A). Similar coupling approaches have already been implemented in the production of glycoconjugates [40].

After 24 h, the LNTII titer reached approximately 10 mM, while about 9 mM UDP‐GlcNAc remained unconsumed, indicating a total UDP‐GlcNAc generation of about 19 mM (Figure 6A). This corresponds to roughly 90% recycling of UDP, as the generated UDP released during the glycosyl transfer was effectively converted back into UDP‐GlcNAc, sustaining the reaction. Lower substrate concentrations in parallel experiments resulted in proportionally lower product titers. The reaction plateau suggests that LgtA enzyme activity diminished after 24 h under these conditions, contributing to UDP‐GlcNAc accumulation. The substitution of Mn^2+^ by Mg^2+^ as cofactor and adjustment of pH to 9 influences LgtA activity according to Blixt et al. [41].

In summary, the one‐pot approach efficiently couples the enzymatic synthesis of UDP‐GlcNAc with glycosylation of lactose to LNTII, achieving high titers and substantial nucleotide sugar recycling, which is beneficial for process economy and scalability.

#### 2.5.2 LNnT

For LNnT synthesis, LNTII and UDP‐Gal were used in crude form, prepared by ultrafiltration (10 kDa) to remove enzymes, without any further chromatographic or specific purification.

**FIGURE 7:**
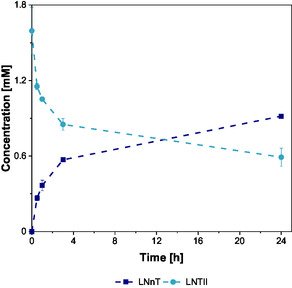
Synthesis of LNnT using noncommercial LNTII and UDP‐Gal. Experimental conditions: 150 mM MOPS (pH 7), 10 mM MnCl_2_, 1.5 mM LNTII, 1.5 mM UDP‐Gal, 0.2 g/L LgtB. Incubation for 24 h at 37°C and 300 rpm. Enzymatic reactions were performed in biological triplicate, mean and standard deviation.

During the experiment, the addition of MnCl_2_ to the reaction mixture led to the formation of a white precipitate, likely attributed to polyphosphate forming complexes with Mn^2+^ ions. This complexation may have reduced the availability of free Mn^2+^, an essential cofactor for enzymatic activity, potentially impacting the overall reaction efficiency. Despite these conditions, the enzymatic transformation proceeded, yielding a product conversion of LNTII to LNnT of 60% and a LNnT titer of 1 mM after 24 h (Figure 7).

## 3 Conclusion

High cost and low availability of UDP‐GlcNAc and UDP‐Gal hinder the large‐scale enzymatic synthesis of valuable glycans such as HMOs. To reduce the biocatalyst workload, an *E. coli* strain was developed here, producing all enzymes required for both multienzyme cascades simultaneously. Using UMP and GlcNAc or Gal as substrates, the purified enzyme mixture achieved conversion yields reaching 100%. Additionally, using anion‐exchange chromatography, UDP‐GlcNAc and UDP‐Gal were purified to 85–99% UV purity in milligram quantities. The nucleotide synthesis cascade was successfully integrated as a UDP‐GlcNAc regeneration system with a Leloir glycosyltransferase for the enzymatic synthesis of LNTII. This integrated approach eliminates the need for intermediate purification of nucleotide sugars and holds potential for cost‐effective, large‐scale in vitro HMO synthesis. Furthermore, LNTII can be easily extended to LNnT by using crude UDP‐Gal obtained via a simplified ultrafiltration‐based purification process.

## 4 Experimental Section

### 4.1 Recombinant Enzyme Production

#### 4.1.1 Multienzyme Cascades

Cell cultivation was carried out in complex Terrific Broth (TB) medium with different antibiotics (Table 4). Recombinant enzymes were expressed using the standard T7 expression protocol and purified by immobilized metal affinity chromatography (IMAC). Purified protein samples and crude cell lysate were stored in 50% (v/v) glycerol at −20°C until usage. Purity of the protein samples was determined by SDS‐PAGE using the ImageJ software (National Institutes of Health, USA).

**TABLE 4:** Duet vector resistance marker and corresponding used antibiotics concentration.

Duet vector	Antibiotic	Concentration, µg/mL
**pET‐Duet**	Ampicillin	50
**pCDF‐Duet**	Streptomycin	50
**pRSF‐Duet**	Kanamycin	30
**pACYC‐Duet**	Chloramphenicol	34

#### 4.1.2 Leloir Glycosyltransferases

For the enzymatic synthesis of LNTII, the β−1,3‐*N*‐acetylglucosaminyltransferase LgtA from *Neisseria meningitidis* was expressed in *E. coli* BL21(DE3). The gene was cloned into the pET‐26b(+) vector with N‐terminal His–SUMO tag to improve solubility and enable purification via IMAC. The protein was expressed using a standard T7 expression protocol in TB media.

For the synthesis of LNnT, the β−1,4‐galactosyltransferase LgtB from *Neisseria meningitidis* was expressed. The lgtB gene was cloned into the pET‐15b vector to generate a construct with N‐terminal His‐tag. Protein expression using a standard T7 expression protocol was carried out in Luria–Bertani (LB) medium supplemented with 0.5 M NaCl.

Both glycosyltransferases were purified by IMAC and stored in 50% (v/v) glycerol at −20°C.

### 4.2 Enzymatic Reactions

Unless otherwise specified, multienzyme cascades were conducted in 500 µL of 150 mM Tris‐HCl buffer (pH 9) at 37°C and 300 rpm for 24 h. Cosubstrate concentrations were 25 mM PolyP*_n_* and 2.5 mM ATP, with a total protein concentration of 1 g/L and a cofactor concentration of 75 mM MgCl_2_.

To couple the UDP‐GlcNAc and the LNTII synthesis, the reaction was conducted in 500 µL of 150 mM Tris‐HCl buffer (pH 9) at 37°C and 500 rpm for 24 h. Substrate concentrations were 10 mM UMP, 50 mM GlcNAc, and 100 mM lactose. Additionally, 45 mM PolyP*_n_*, 150 mM MgCl_2_, 2.5 mM ATP, 1 g/L crude cell lysate, and 0.2 g/L purified LgtA were applied.

For the synthesis of LNnT, LNTII and UDP‐Gal from previous reaction mixtures were ultrafiltered using an Amicon Ultra centrifugal filter units (Merck, DE) with a 10 kDa cutoff. The reaction with a total volume of 200 µL was set up with starting concentrations of 1.5 mM LNTII, 1.5 mM UDP‐Gal, and 10 mM MnCl_2_ in 150 mM MOPS (pH 7). The concentration of purified LgtB was 0.2 g/L. Incubation took place at 37°C and 300 rpm for 24 h.

### 4.3 Chromatographic Purification of UDP‐GlcNAc and UDP‐Gal

An anion‐exchange chromatographic method was employed to purify UDP‐GlcNAc and UDP‐Gal. Starting from the crude reaction mixture proteins were first removed using an Amicon Ultra centrifugal filter units (Merck, DE) with a 10 kDa cutoff. For purification, a setup consisting of a 5 mL HiTrap Q HP column (Cytiva, DE) and an Äkta pure 25 device (GE Health, SE) was used. A 1 mM NaOH aqueous system with 1 M NaCl serving as eluent was used.

### 4.4 Quantification with High‐Performance Anion‐Exchange Chromatography

**FIGURE 8:**
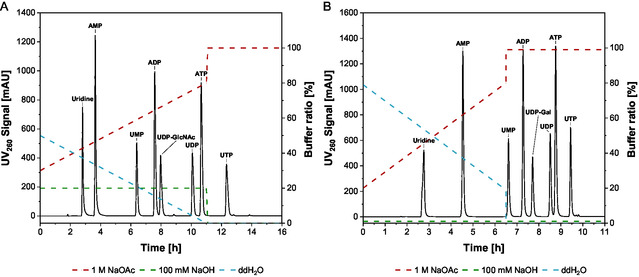
UV signal at 260 nm from the chromatographic separation of a 150 µM standard calibration solution via HPAEC. (A) Gradient elution for UDP‐GlcNAc standard solution. (B) Gradient elution for UDP‐Gal standard solution.

For quantification, reaction samples were quenched with 90°C hot water and analyzed by high‐performance anion‐exchange chromatography (HPAEC) and UV absorbance was measured at a wavelength of 260 nm. A setup of CarboPac PA200 analytical & guard column (Thermo Fisher Scientific, USA) with the HPAEC unit ICS‐5000+ (Thermo Fisher Scientific, USA) was used. The buffer system consisted of 100 mM NaOH and deionized water, with 1 M NaOAc serving as the eluent. To achieve optimal peak separation of UDP‐GlcNAc, the column temperature was maintained at 30°C, with a gradient as shown in Figure 8A, and a flow rate of 0.5 ml/min. For the detection of UDP‐Gal, the column temperature was increased to 40°C, utilizing the gradient depicted in Figure 8B.

**FIGURE 9:**
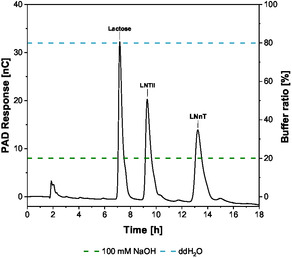
PAD response of the chromatographic separation of a 10 µM standard calibration solution using HPAEC.

Carbohydrates were monitored separately by a pulsed amperometric detection (PAD) detector using an isocratic elution at a flow rate of 0.5 mL/min and a column temperature of 30°C (Figure 9).

### 4.5 Nuclear Magnetic Resonance (NMR)

^1^H and ^13^C‐NMR spectra were recorded at 600 MHz on a Bruker Avance 600 in D_2_O. Chemical shifts for NMR are reported as parts per million (ppm).

## Supporting Information

Additional supporting information can be found online in the Supporting Information section.

## Author Contributions

**Tuan Son Hoang**: conceptualization (lead), formal analysis (lead), investigation (lead), methodology (lead), project administration (lead), supervision (lead), validation (lead), writing – original draft (lead), writing – review and editing (lead). **Fabian Lange**: investigation (supporting). **Sebastian Bruno Kleeberg**: investigation (supporting). **Nam‐Hai Hoang**: investigation (supporting), methodology (supporting), visualization (supporting). **Udo Reichl**: project administration (supporting), resources (lead), supervision (lead), writing – review and editing (supporting). **Thomas F. T. Rexer**: conceptualization (lead), funding acquisition (lead), methodology (supporting), project administration (supporting), supervision (lead), writing – review and editing (lead).

## Funding

This work was supported by Bundesministerium für Wirtschaft und Klimaschutz (Grant 03EFRST036).

## Conflicts of Interest

The authors declare the following financial interests/personal relationships which may be considered as potential competing interests: Udo Reichl reports a relationship with glyxera GmbH that includes: board membership and equity or stocks. Udo Reichl reports a relationship with eversyn GmbH that includes: board membership and equity or stocks. Thomas Rexer reports a relationship with eversyn that includes: board membership, employment, and equity or stocks. Nam‐Hai Hoang reports a relationship with eversyn that includes: board membership and equity or stocks. If there are other authors, they declare that they have no known competing financial interests or personal relationships that could have appeared to influence the work reported in this paper.

## Supporting information

Supplementary Material

## Data Availability

Research data are not shared.
