# Supplementary material for: Multienzyme Platform for the Synthesis of UDP Sugars and Human Milk Oligosaccharides
Source: Chembiochem. 2026 Apr 22;27(8):e202500716. doi: 10.1002/cbic.202500716 (PMC13101873; doi:10.1002/cbic.202500716)
Supplement: Supplementary file 1 — Supplementary Material [file CBIC-27-e202500716-s001.pdf]

Supporting Information

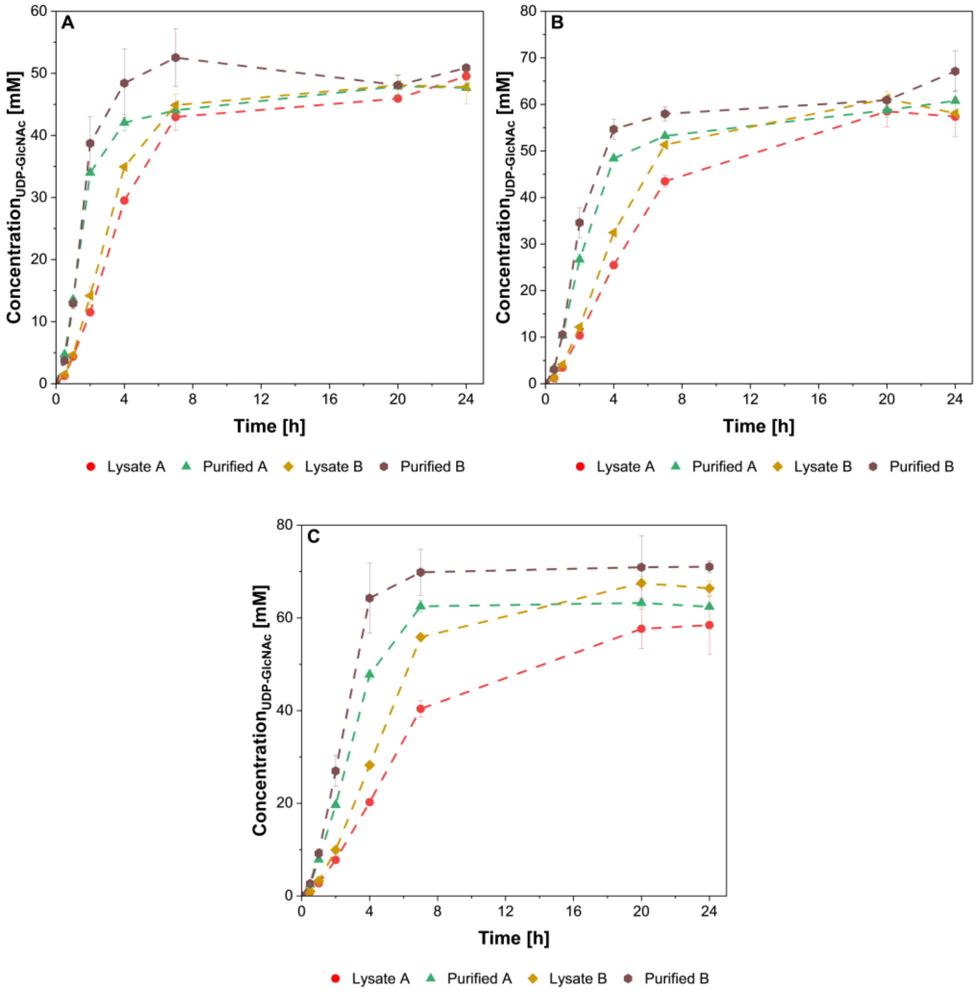

Figure SI 1. Time course of the synthesis of UDP-GlcNAc using crude cell lysates (Lysate A and B) and purified enzyme compositions (Purified A and B) of strain A and B. (A) synthesis using 50 mM UMP and GlcNAc. (B) synthesis using 70 mM UMP and GlcNAc. (C) synthesis using 100 mM UMP & GlcNAc. Enzymatic reactions were performed in biological triplicate; mean and standard deviation.

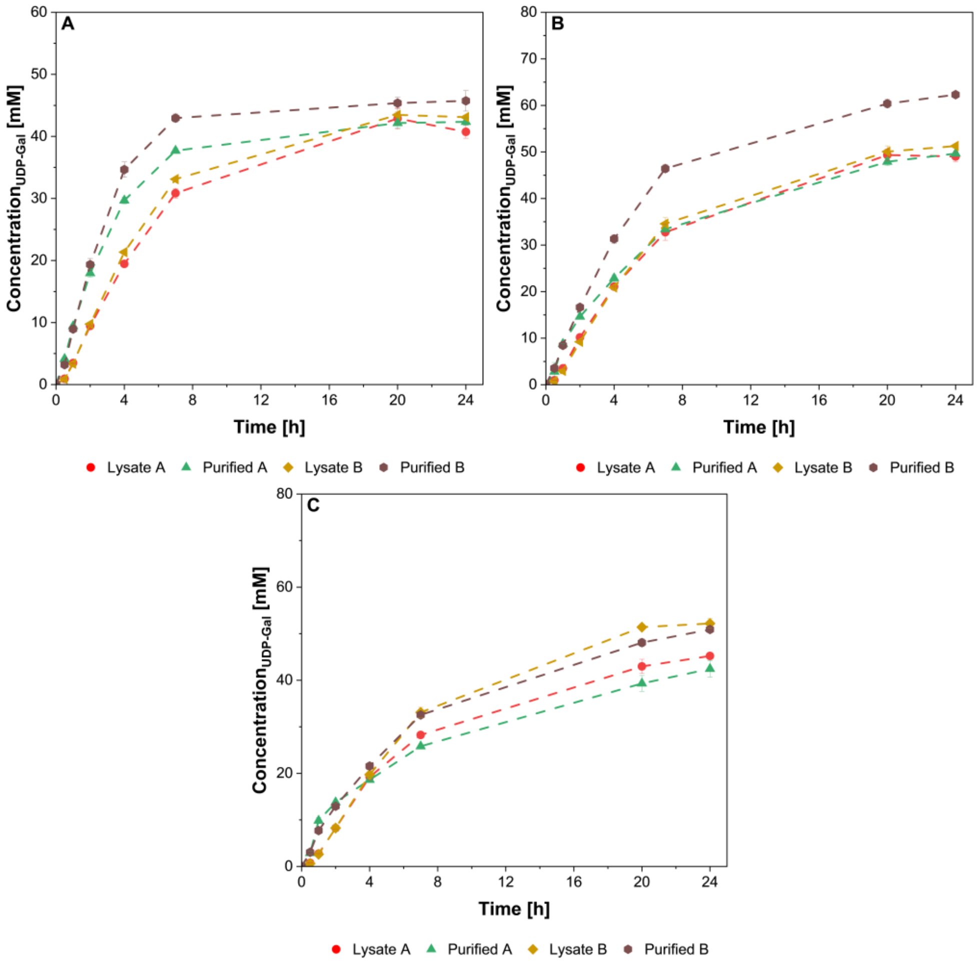

Figure SI 2. Time course of the synthesis of UDP-Gal using crude cell lysates (Lysate A and B) and purified enzyme compositions (Purified A and B) of strain A and B. (A) synthesis using 50 mM UMP and Gal. (B) synthesis using 70 mM UMP and Gal. (C) synthesis using 100 mM UMP & Gal. Enzymatic reactions were performed in biological triplicate; mean and standard deviation.

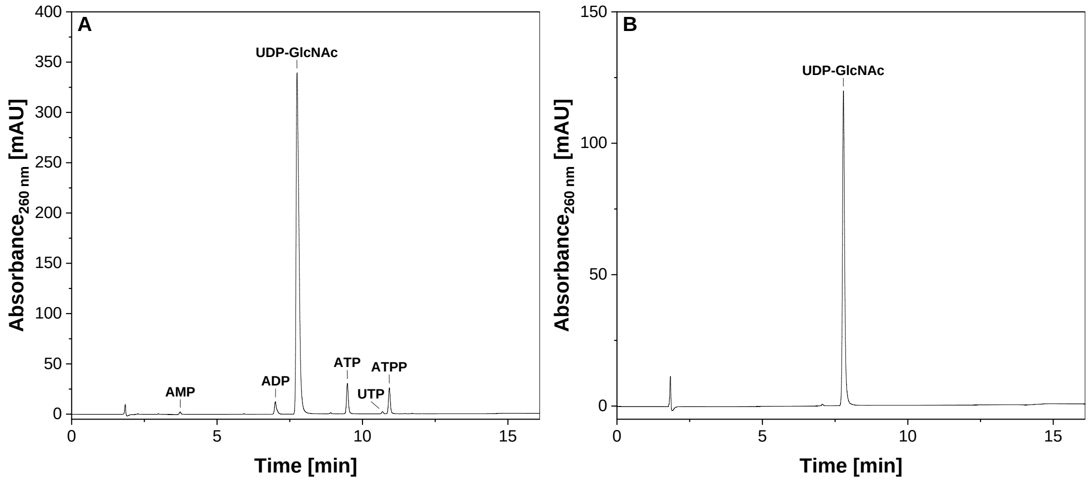

Figure SI 3. HPAEC chromatograms of samples from the purification of UDP-GlcNAc by anion-exchange chromatography. Purity of samples refers to the UV spectrum. (A) Absorbance of the reaction mixture before purification at 95 % purity. (B) Fraction with >99 % purity after purification.

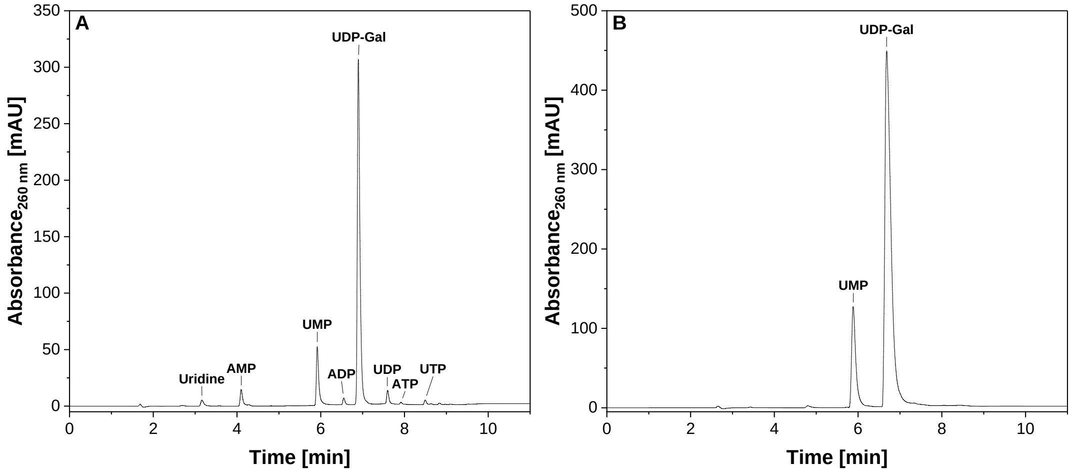

Figure SI 4. HPAEC chromatograms of samples from the purification of UDP-Gal by anion-exchange chromatography. Purity of the samples refers to the UV spectrum. (A) Absorbance of the reaction mixture before purification at 78 % purity. (B) Fraction with >85 % purity after purification.

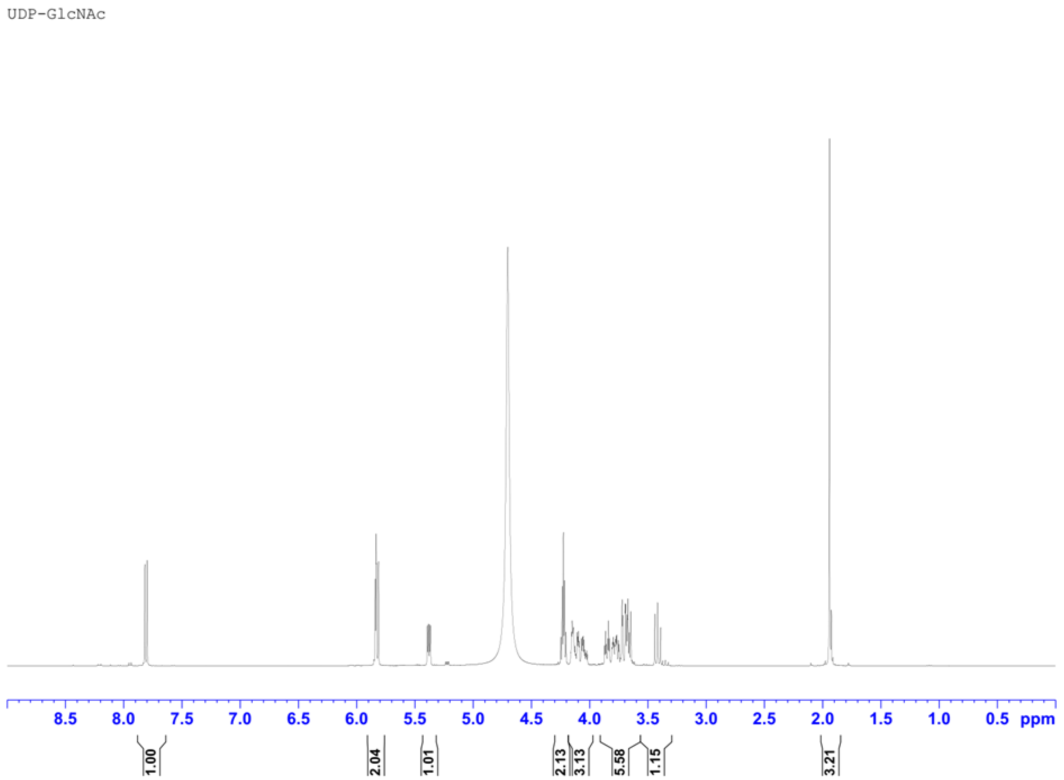

Figure SI 5. ^{1}H-NMR spectrum of the purified UDP-GlcNAc.

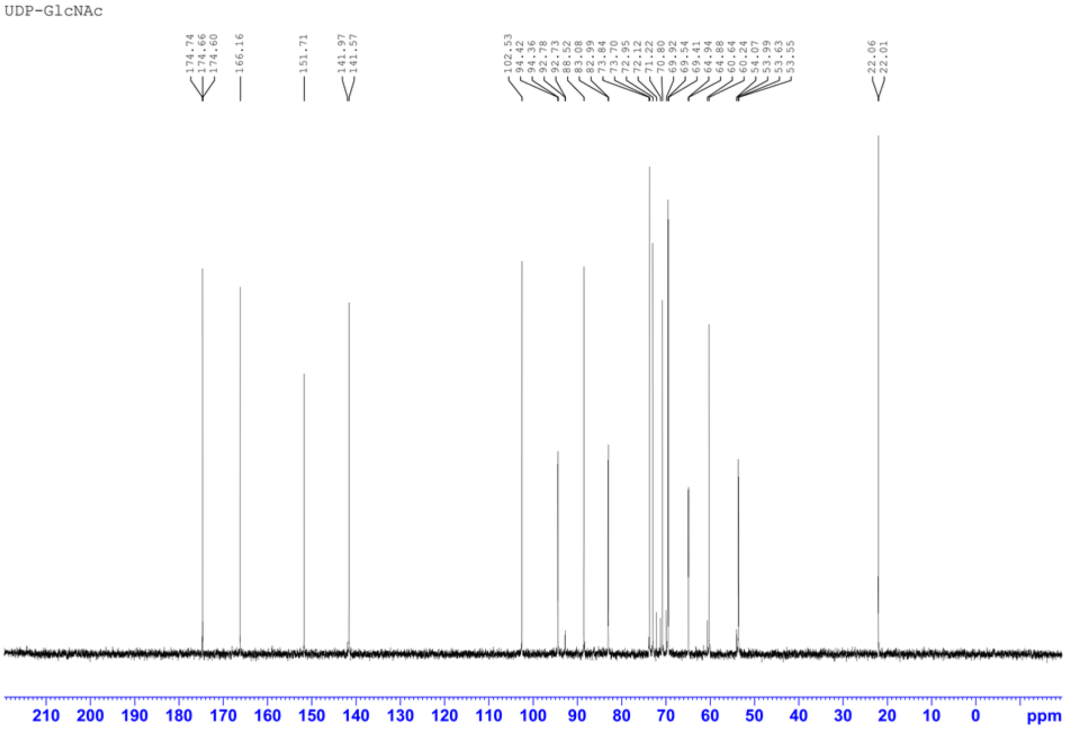

Figure SI 6. ^{13}C NMR spectrum of the purified UDP-GlcNAc.

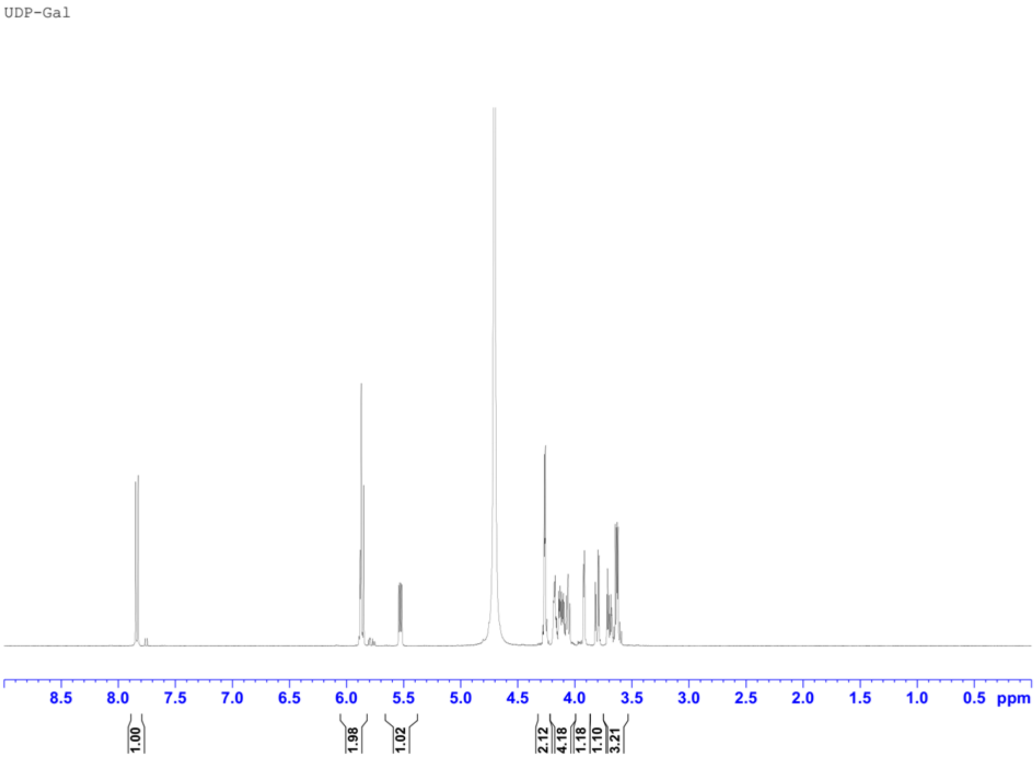

Figure SI 7. ^{1}H-NMR spectrum of the purified UDP-Gal.

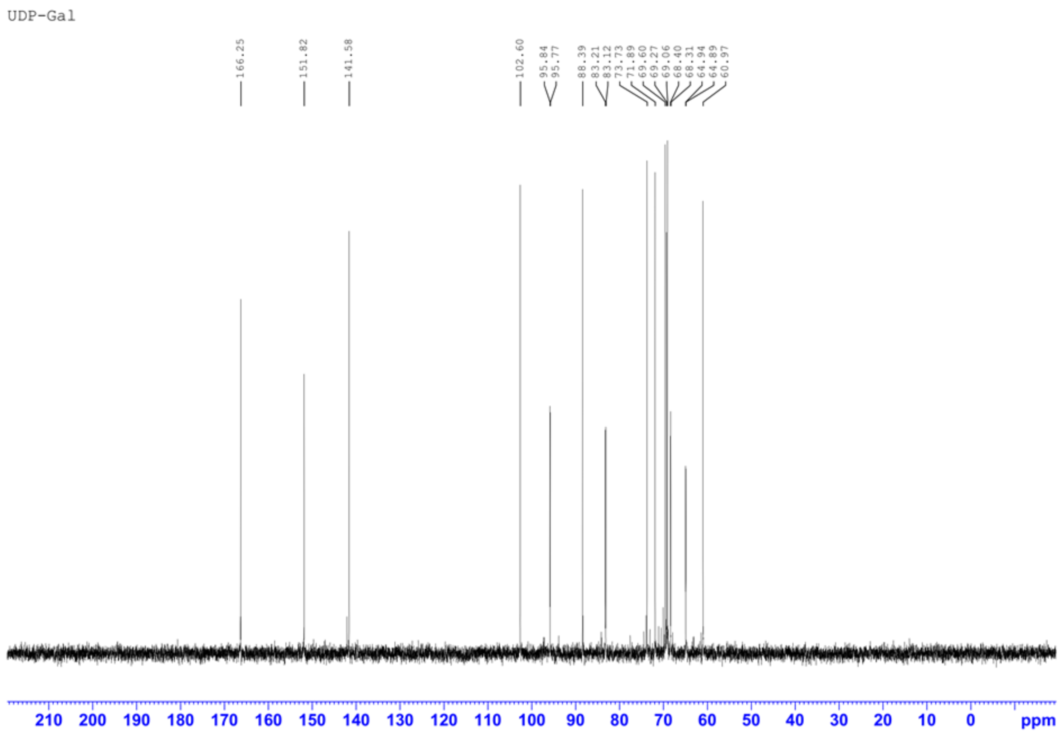

Figure SI 8. ^{13}C NMR spectrum of the purified UDP-Gal.

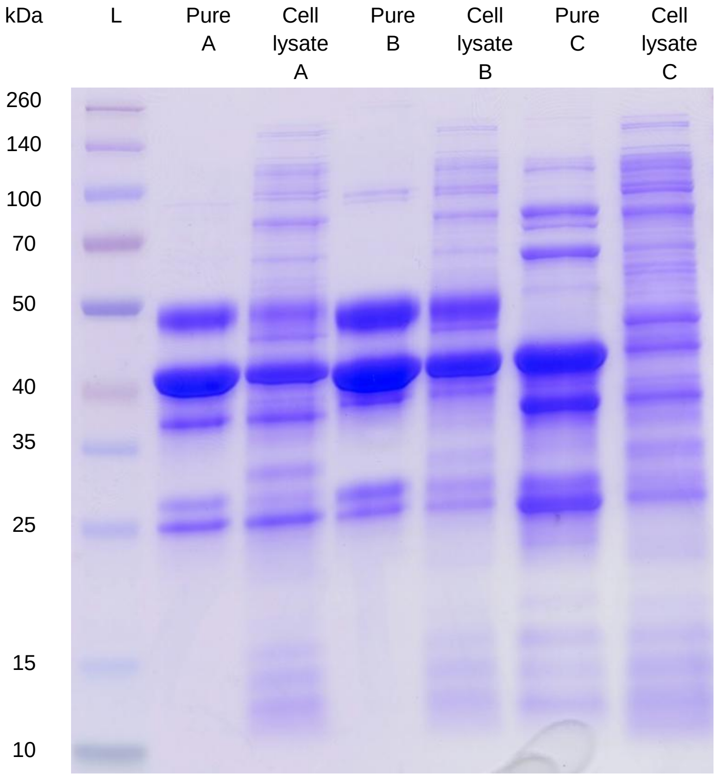

Figure SI 9. SDS-PAGE of the purified enzyme compositions (Pure A, B and C) and the crude cell lysates (Cell lysate A, B and C) of the corresponding expression strains. About 2 μg of protein was loaded per well.
